# Metabolic Dysregulation, Inflammation, and Median Nerve Dysfunction in Patients with Type 2 Diabetes Mellitus with Carpal Tunnel Syndrome

**DOI:** 10.3390/ijms27114995

**Published:** 2026-05-30

**Authors:** Adina Stoian, Simona Cernea, Claudia Bănescu, Mircea Stoian, Andrei Manea, Florina Gliga, Dumitru Golban, Andrei Stîngaciu, Rodica Bălașa

**Affiliations:** 1 Department of Pathophysiology, George Emil Palade University of Medicine, Pharmacy, Science, and Technology of Târgu Mureș, Gheorghe Marinescu Street No. 38, 540142 Târgu Mureș, Romania; adina.stoian@umfst.ro (A.S.); florina.gliga@umfst.ro (F.G.); 2 1st Neurology Clinic, Mureș County Clinical Emergency Hospital, 540136 Târgu Mureș, Romania; golban.dumitru2000@gmail.com (D.G.); rodica.balasa@umfst.ro (R.B.); 3 Department M3/Internal Medicine I, George Emil Palade University of Medicine, Pharmacy, Science and Technology of Târgu Mureş, 540142 Târgu Mureş, Romania; simona.cernea@umfst.ro; 4 Diabetes, Nutrition and Metabolic Diseases Outpatient Unit, Emergency County Clinical Hospital, 540136 Târgu Mureş, Romania; 5 Genetics Department, Center for Advanced Medical and Pharmaceutical Research, George Emil Palade University of Medicine, Pharmacy, Science and Technology of Târgu Mureș, Gheorghe Marinescu Street No. 38, 540136 Târgu Mureș, Romania; claudia.banescu@umfst.ro; 6 Department of Anesthesiology and Intensive Care Medicine, George Emil Palade University of Medicine, Pharmacy, Science and Technology of Târgu Mureș, 540103 Târgu Mureș, Romania; mircea.stoian@umfst.ro; 7 Doctoral School of Medicine and Pharmacy, George Emil Palade University of Medicine, Pharmacy, Science, and Technology of Târgu Mureș, Gheorghe Marinescu Street No. 38, 540142 Târgu Mureș, Romania; 8 Department of Radiology, George Emil Palade University of Medicine, Pharmacy, Science, and Technology of Târgu Mureș, Gheorghe Marinescu Street No. 38, 540142 Târgu Mureș, Romania; 9 Intensive Care Unit, Mures Clinical County Hospital, Street Gheorghe Marinescu No. 1, 540136 Târgu Mureș, Romania; andreistingaciu@yahoo.com; 10 Department of Neurology, George Emil Palade University of Medicine, Pharmacy, Science and Technology of Târgu Mureș, 540142 Târgu Mureș, Romania

**Keywords:** carpal tunnel syndrome, type 2 diabetes mellitus, chronic inflammation, nerve conduction studies, monocytes

## Abstract

Carpal tunnel syndrome (CTS) is the most common compressive mononeuropathy. In patients with type 2 diabetes mellitus (T2DM), chronic hyperglycemia, microangiopathy, and systemic inflammation increase the vulnerability of peripheral nerves to compression. This study aimed to assess the relationship between CTS severity and clinical, metabolic, inflammatory, and electrophysiological parameters in patients with T2DM. A cross-sectional study was conducted from June 2023 to June 2024, involving patients diagnosed with T2DM. Electrophysiological assessment of the upper and lower limbs was performed using a four-channel electromyography apparatus. Clinical and anthropometric data and laboratory parameters were obtained, as well as the results of nerve conduction studies (NCS). One hundred and twenty-three patients with T2DM were included in the study. The prevalence of moderate-to-severe forms of CTS was 43.9%, and bilateral involvement was present in 21.95% of patients. Patients with moderate-to-severe CTS had significantly higher hemoglobin A1c (HbA1c) (*p* = 0.004), glycemia (*p* < 0.001), and Triglyceride–Glucose Index (*p* = 0.018) compared with those without CTS/with mild forms. The number of monocytes was significantly higher in the group with moderate-to-severe forms (*p* = 0.012), suggesting a chronic inflammatory state. In the logistic regression analysis, hemoglobin HbA1c emerged as an independent predictor of CTS severity, with each 1% increase associated with approximately a 60% higher risk of moderate/severe CTS. NCS analysis showed significant correlations between median nerve parameters and those of the lower-limb peripheral nerves, particularly the tibial and sural nerves, suggesting an association with generalized diabetic peripheral neuropathy. Professional activity was significantly associated with moderate-to-severe CTS (OR = 3.5). CTS is a common complication in patients with T2DM and is associated with worse glycemic control, insulin resistance, systemic inflammation, and peripheral neuropathic damage.

## 1. Introduction

Carpal tunnel syndrome (CTS) is the most common entrapment mononeuropathy and results from compression of the median nerve at the carpal tunnel due to increased pressure at this site [1]. Its presence can lead to disability, reduced work capacity, and a substantial socioeconomic burden [2,3].

Damage to the vascular endothelium can increase the vascular permeability, causing local swelling, which can lead to compression of the structures at this level, including the median nerve [2,4]. Damage to the microvascular system can also make the nerve more vulnerable to mechanical stress, initially leading to demyelination and eventually secondary axonal degeneration [2,4,5].

The CTS manifests through symptoms like paresthesia, numbness, and neuropathic pain in the affected nerve area [6] that worsen as the disease progresses, followed by motor symptoms with atrophy of the thenar muscles [7]. Symptoms often first appear at night and thereafter during the day. Risk factors for CTS include repetitive vibration work, endocrinopathies (like hypothyroidism), obesity, pregnancy, rheumatologic conditions, and diabetes [1,8]. Without proper diagnosis and treatment, it tends to worsen over time, leading to permanent loss of sensation and motor function in the affected hand [1].

Several studies have examined the link between Type 2 diabetes mellitus (T2DM) and CTS, but their findings have been inconsistent. While it is established that T2DM leads to diabetic peripheral neuropathy, it remains unclear how much T2DM is also involved in the development of compression neuropathies like CTS [9,10,11]. Some studies estimate the prevalence of CTS at 14% among people with diabetes [12], and about 30% in the presence of diabetic polyneuropathy [12]. Evaluation and treatment require a collaborative effort from an interdisciplinary medical team [1].

Nerve conduction studies (NCS), sometimes combined with needle electromyography (EMG), have improved the accuracy of confirming CTS. NCS is a highly sensitive tool for diagnosing CTS and is frequently used for this purpose [13]. We conducted this study to investigate the factors associated with CTS in T2DM patients—including anthropometric and laboratory parameters, such as inflammatory and metabolic biomarkers—and the electrophysiological changes in the median nerve compared with those in other sensory and motor nerves.

## 2. Results

A total of 154 patients with T2DM were screened, of whom 31 met the exclusion criteria. Ultimately, 123 patients were included in the analysis (58 females and 65 males). Their ages ranged from 40 to 84 years (mean ± standard deviation (SD) 67.94 ± 8.8 years). The median duration of T2DM was 12 years (interquartile range (IQR), 6–20 years).

When minor (including subclinical) forms of CTS were included, the prevalence of CTS in our study was 91.05% (112 patients). Of these, 58 patients (47.2%) had mild CTS, and 54 patients (43.9%) had moderate-to-severe CTS. Bilateral moderate-to-severe CTS was present in 27 patients (21.95%). Eleven patients (8.95%) had no evidence of CTS. Forty-four patients had moderate to severe CTS in the right hand, and 37 in the left hand (Table S1).

In subsequent analyses, patients with moderate-to-severe CTS (CTS+) were compared with those without CTS or with mild CTS (CTS−) to reduce the potential influence of subclinical or mild electrophysiological abnormalities and to better capture clinically meaningful nerve dysfunction in patients with T2DM.

The general characteristics of the patients included in the study are presented in Table 1. The comparison of clinical and demographic parameters among patients without CTS/with mild CTS and with moderate/severe CTS showed no statistically significant differences in most variables examined, including age, body mass index (BMI), duration of T2DM, blood pressure (BP) levels, antidiabetic treatments, and socio-demographic factors.

**Figure 1:**
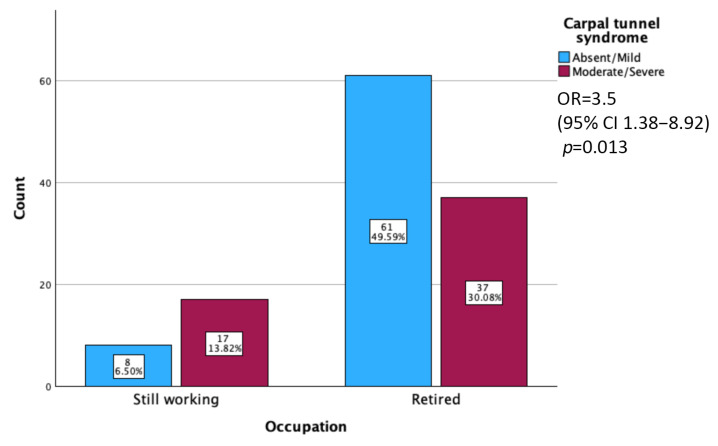
Distribution of patients based on occupational status and severity of carpal CTS.

**Figure 2:**
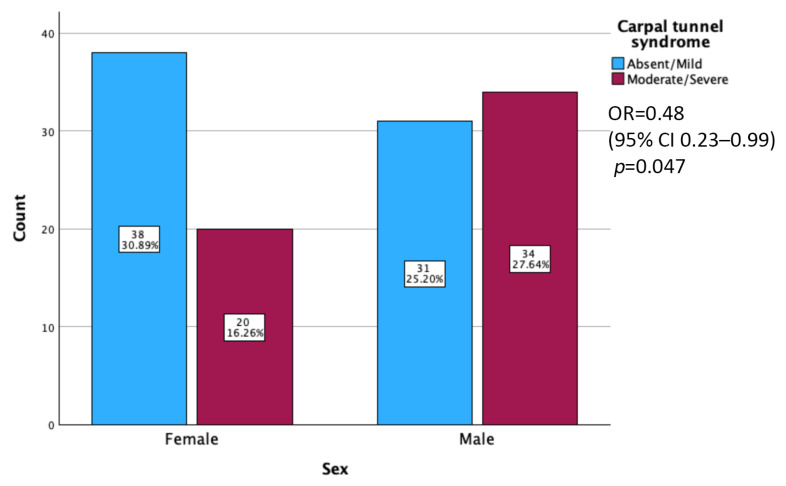
Distribution of patients by gender and severity of CTS.

Statistically significant differences were observed in active professional status and gender (Table 1). Professionally active patients presented a significantly increased risk of moderate/severe CTS (31.4% vs. 11.5%; odds ratio (OR) = 3.5, [95% confidence interval (CI): 1.38−8.92], *p* = 0.013) (Figure 1). Also, female gender was associated with a lower probability of moderate/severe CTS (OR = 0.48 [95%CI: 0.23–0.99], *p* = 0.047) (Figure 2). BMI and the abdominal circumference were slightly higher in the CTS+ group, but did not reach statistical significance (BMI: *p* = 0.053; abdominal circumference: *p* = 0.065).

Table 2 indicates the biological, metabolic, and inflammatory markers in patients with absent/mild CTS and in those with moderate/severe CTS. In the comparison between patients with absent/mild CTS and those with moderate/severe CTS, significant differences were found for glycemic and inflammatory markers. The group with moderate/severe CTS had higher hemoglobin A1c (HbA1c) (7.44% vs. 6.83%; *p* = 0.004), glycemia (150 vs. 120 mg/dL; *p* < 0.001), and Triglyceride–Glucose Index (TyG) (9.37 ± 0.74 vs. 9.06 ± 0.63; *p* = 0.018). Additionally, monocyte count was significantly higher in the moderate/severe CTS group (0.70 vs. 0.56; *p* = 0.012), while Platelet-to-lymphocyte ratio (PLR) was lower (*p* = 0.009).

A multivariable binary logistic regression analysis examining the relationship between sex, HbA1c, and BMI and the presence of CTS was statistically significant (χ^2^(3) = 19.51, *p* < 0.001), indicating that the predictors distinguished patients with moderate-to-severe CTS from those without CTS or with mild CTS. The model explained approximately 20.1% of the variance in CTS status (Nagelkerke R^2^ = 0.201).

Among the independent variables, only HbA1c was a statistically significant predictor (OR = 1.60, 95% CI: 1.199–2.134, *p* = 0.001). This indicates that each 1% increase in HbA1c is associated with a 60% rise in the odds of CTS. Sex and BMI approached significance (*p* = 0.057 and *p* = 0.076, respectively), with trends suggesting possible associations warranting further research. The model was internally validated using 1000 bootstrap samples. The prediction model formula was:

$$\ln\frac{p}{1-p}=-6.262+0.737\cdot Sex+0.068\cdot BMI+0.470\cdot HbA1c$$

where *p* is the probability of CTS.

**Figure 3:**
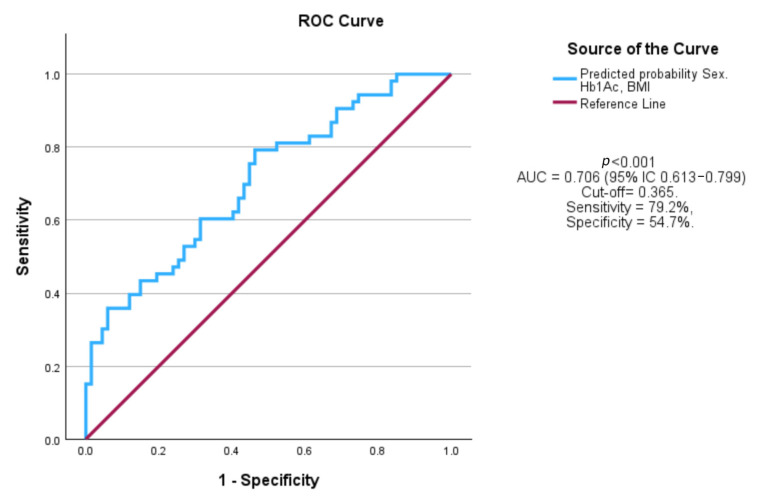
ROC curve of the logistic model for predicting CTS.

The model’s discriminative ability was evaluated using the receiver operating characteristic (ROC) curve (Figure 3). The area under the curve (AUC) was 0.706 (95% CI: 0.613–0.799; *p* < 0.001), indicating acceptable differentiation between individuals with and without CTS. Using the Youden Index, the optimal probability threshold was determined to be 0.356, yielding a sensitivity of 79.2% and a specificity of 53.7%.

In an additional multivariable binary logistic regression model, age category was included as a categorical predictor, with age category 1 (under 60) serving as the reference group. The model remained statistically significant, with χ^2^(6) = 22.332, *p* = 0.001, and a Nagelkerke R^2^ of 0.227. However, age category did not independently predict moderate-to-severe CTS (Wald χ^2^(3) = 2.646, *p* = 0.450). None of the other age groups showed a significant association with CTS severity compared to the reference: age category 2 (60–65), OR = 0.341, 95% CI: 0.083–1.405, *p* = 0.136; age category 3 (66–70), OR = 0.706, 95% CI: 0.198–2.510, *p* = 0.590; and age category 4 (over 70), OR = 0.911, 95% CI: 0.310–2.681, *p* = 0.866. HbA1c was the only remaining statistically significant independent predictor, with OR = 1.688, 95% CI: 1.239–2.300, *p* < 0.001, indicating that the association between HbA1c and CTS severity is independent of age, sex, and BMI. The models results are summarized in Table 3.

The analysis of the electroneurographic parameters of the lower-limb nerves, as shown in Table 4, indicated significantly lower amplitudes in patients with moderate-to-severe carpal tunnel syndrome than in those with absent or mild CTS. Statistically significant differences were found for the amplitude of the left tibial nerve at the ankle (4.5 vs. 5.3 mV; *p* = 0.028), the amplitude of the left sural nerve (7.6 vs. 10.9 μV; *p* = 0.004), and the amplitude of the right sural nerve (7.65 vs. 11 μV; *p* = 0.014).

No significant differences were found for the amplitude of the right tibial nerve, left and right common peroneal nerves (*p* = 0.259, *p* = 0.074, and *p* = 0.088, respectively).

Table 5 displays the correlations between motor and sensory conduction velocities of the median nerve and the compound muscle action potential (CMAP) and sensory nerve action potential (SNAP) amplitudes of the lower-limb nerves.

**Figure 4:**
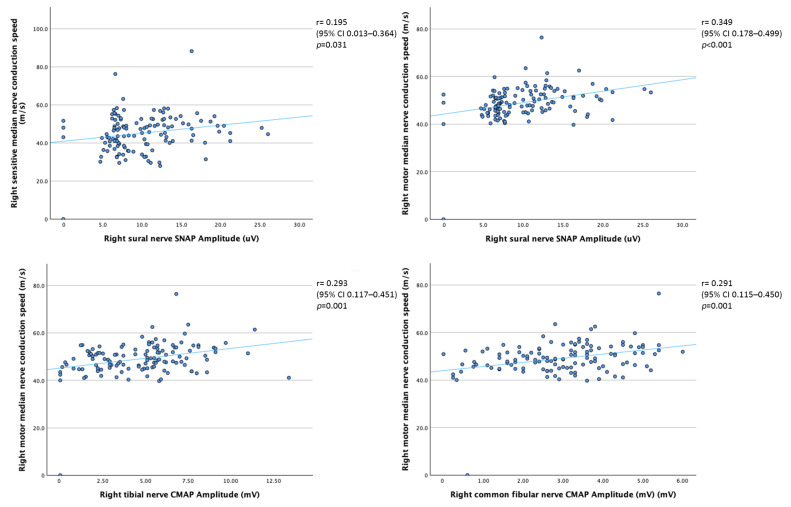
Representation of the linear regression between the amplitude of the right sural, tibial, and common peroneal nerves and the sensory and motor conduction velocity of the right median nerve.

Correlations were observed between the amplitudes of the tibial and sural nerves and the motor conduction velocity of the bilateral median nerve. Higher amplitudes in the nerves of the lower limb were linked to improved median nerve conduction velocity. Specifically, the amplitude of the left tibial nerve exhibited a weak positive correlation with the left (r = 0.378; *p* < 0.001) and right (r = 0.305; *p* = 0.001) median motor conduction velocities. Similarly, the amplitudes of the left and right sural nerves showed significant weak positive correlations with the bilateral median motor conduction velocity (r = 0.348–0.369; *p* < 0.001). Figure 4 shows a graphical representation of the most important correlations.

For median nerve sensory conduction velocity, correlations were weaker but still statistically significant, especially for the amplitude of the left tibial nerve and bilateral sural nerve (r between 0.182 and 0.226; *p* < 0.05). In contrast, correlations with the common peroneal nerve were weaker and more inconsistent, particularly in the case of the right sensory median nerve (*p* = 0.615).

Correlation matrix analysis using the Spearman test, included in Table 6, revealed significant associations between the electroneurographic parameters of the median nerve and those of other peripheral nerves, supporting the presence of a systemic neurophysiological impairment.

Within the median nerve, a negative weak but significant correlation was observed between motor latency and motor conduction velocity (r = −0.388; *p* < 0.001), as well as a negative strong correlation between sensory latency and sensory conduction velocity (r = −0.601; *p* < 0.001). Median CMAP amplitude also showed a weak positive correlation with motor (r = 0.244; *p* < 0.01) and sensory (r = 0.302; *p* < 0.01) conduction velocity.

The parameters of the median nerve showed significant moderate correlations with those of the ulnar nerve, especially between median motor conduction velocity and ulnar nerve velocity (r = 0.413; *p* < 0.001), as well as with the tibial nerve (r = 0.491; *p* < 0.001) and sural nerve (r = 0.359; *p* < 0.001).

Significant moderate positive correlations were observed between sural nerve SNAP amplitude and median nerve sensory conduction velocity (r = 0.500; *p* < 0.001), as well as between sural nerve velocity and median sensory velocity (r = 0.500; *p* < 0.001). These findings indicate a close link between median nerve injury and distal peripheral neuropathy.

## 3. Discussion

### 3.1. Prevalence of CTS

Numerous studies have examined the link between diabetes and CTS, based on the idea that diabetes increases the vulnerability of peripheral nerves to compression, but the findings have often been inconsistent. There might be a connection between T2DM and CTS in certain subgroups, which would require examining the effects of medication use, the method of patient inclusion, and the possibility that the lack of association could be due to the predominance of cases without severe complications treated in neurology or diabetology outpatient clinics.

The prevalence of CTS ranges from 3% to 6% in the overall adult population [14,15,16]. For example, in Sweden, an annual incidence of 428 cases among women and 182 among men per 100,000 adults has been reported [17]. However, these figures may vary by region and country [17,18]. Furthermore, annual surgeries for CTS are more frequent in Sweden and the United States than in the United Kingdom [16,17,19].

Studies examining the link between T2DM and CTS show significant variation across geographical areas and study designs. The association tends to be weaker in population-based studies compared to hospital-based studies [20,21,22]. The study by Jason Low et al. (2021), for example, has not found differences in CTS risk among patients with T2DM [23]. Another retrospective case–control study conducted at a single center in the Netherlands did not identify T2DM as a predictor of CTS (OR: 0.99; 95% CI: 0.66–1.47) after adjusting for age, sex, and BMI [24]. A study conducted at a university hospital in Turkey indicated that the risk of developing CTS was 60 times higher in patients with diabetes than in controls (OR: 60; 95% CI: 13–246) [21].

The reported prevalence of diabetic neuropathy ranged from 30 to 50% among individuals with diabetes, and increases with the length of the disease [16,25]. It is often present at diagnosis, and men are more likely to develop neuropathy [26]. The most common type is symmetric distal polyneuropathy, a significant cause of diabetic foot complications [27].

The prevalence of electrophysiologically confirmed CTS (including mild forms) in our cohort was 91.05%, and moderate-to-severe CTS was present in 43.9% of patients, a prevalence similar to previously reported values. The relatively high prevalence observed in our cohort (including mild forms) compared with previous reports might be explained by several factors. First, the study included only patients with T2DM evaluated in a tertiary care setting, many of whom had long-standing disease and a potentially higher burden of metabolic complications. Second, all patients underwent systematic electrophysiological evaluation with nerve conduction studies, which likely increased the sensitivity to detect mild or subclinical median nerve abnormalities. Therefore, some patients may have shown only mild electrophysiological changes without overt clinical symptoms. The higher overall prevalence observed in our cohort, when mild forms were included, may be attributable to the characteristics of the study population and methodology. To minimize this potential limitation and reduce overestimation due to subclinical cases, we compared patients with moderate-to-severe CTS with those without CTS or with mild CTS, aiming to better capture clinically relevant nerve dysfunction. Discrepancies between studies may also arise from the limited use of NCS in diagnosing CTS, despite its sensitivity and specificity. Failing to consider important covariates can also lead to errors in analysis. Many studies have not correlated with the presence of diabetic peripheral neuropathy, ignoring the fact that its presence can sometimes impact the median nerve [9].

### 3.2. Etiology and Biomarkers of CTS

The trunk of a peripheral nerve is a delicate structure with components that respond differently to trauma [16]. The myelinated and unmyelinated nerve fibers are organized into bundles surrounded by the perineurium, which provides mechanical and chemical protection and helps form the blood-nerve barrier [16]. Inside, the endoneurium individually encloses each nerve fiber (the axon and Schwann cells). The epineurium is the outermost layer that encloses the nerve bundles by embedding them in loose connective tissue [16]. The nerve receives blood supply from small vessels that penetrate these compartments; the vessels in the endoneurium, mostly capillaries, are relatively resistant to trauma, while those in the epineurium are more vulnerable, with damage leading to edema and subsequent fibrosis [16,28].

The exact etiology of CTS is not fully understood, but it is believed to result from compression of the median nerve within the carpal tunnel, which causes ischemia and subsequent segmental demyelination.

Median nerve neuropathy is often a complication of diabetes mellitus, marked by a reduction in myelinated nerve fibers and endoneurial capillaries [29]. Myelinated fibers are more susceptible to nerve compression than unmyelinated ones [20]. In T2DM, hyperglycemia causes intracellular accumulation of sorbitol in neurons and Schwann cells, leading to axonal degeneration and segmental demyelination, thereby increasing the nerve’s vulnerability to compression and promoting the development of CTS [20]. It is believed that diabetes mellitus exacerbates nerve ischemia through advanced glycation end-products, particularly under conditions of chronic oxidative stress driven by increased mitochondrial reactive oxygen species production, fluid buildup and edema, heightened inflammatory cytokine production, and alterations in myofibroblasts [20,23,30]. The mechanisms by which elevated BMI increases the risk of CTS are thought to involve excess adipose tissue within the carpal tunnel, which can lead to progressive narrowing and elevated intracanal pressure [30]. The microcirculation at the level of the median nerve is affected by increased pressure, ultimately leading to axonal loss [30,31].

Although BMI and abdominal circumference tended to be higher in patients with moderate-to-severe CTS, these differences did not reach statistical significance in our cohort and were therefore not further analyzed [32].

Monocytes are a primary source of inflammatory cytokines and are central to the initiation and maintenance of systemic inflammation associated with diabetes [33]. Chronic high blood sugar levels alter gene expression and monocyte behavior. Under these conditions, Toll-like receptor 4 expression increases on monocytes, enhancing the inflammatory response in tissues [34]. Additionally, the chemokine Monocyte Chemoattractant Protein-1 (MCP-1), secreted by monocytes and other inflammatory cells, plays a key role in recruiting and activating monocytes at sites of inflammation [35]. Elevated MCP-1 levels, observed in both early and late stages of T2DM, encourage monocyte infiltration into tissues and contribute to the development of diabetic neuropathy [34]. Inflammatory processes are further amplified by activation of the Nuclear Factor kappa B pathway and increased expression of adhesion molecules like Intercellular Adhesion Molecule 1, which facilitates monocyte and macrophage infiltration into nerves [34,36]. Furthermore, M1 macrophages can impair pancreatic cell function, worsen hyperglycemia, and sustain the cycle of inflammation and metabolic dysregulation [37].

Among the hematological markers in our study, the presence of moderate-to-severe CTS was significantly associated with higher monocyte counts (*p* = 0.012) and lower platelet counts (*p* = 0.009). Other studies investigating the presence of CTS in various pathologies did not find correlations with monocyte or platelet levels [38]. Another study in Turkey involving 407 patients with CTS and 206 subjects without CTS showed a positive correlation between the severity of CTS and NLR (r = 0.224; *p* < 0.001), BMI (r = 0.251; *p* < 0.001), and age (r = 0.333; *p* < 0.001), but no correlations were found with platelet or macrophage counts [39]. A meta-analysis including 7 studies and 365 patients evaluated the effect of local platelet-rich plasma treatment in CTS. It reported a beneficial impact on symptoms, with a tendency toward reduced pain and improved function among treated patients compared with conventional treatment during the first months after the intervention. However, there were no statistically significant differences in the medium term regarding electrophysiological parameters [40].

NLR is a marker of inflammation and is associated with diabetic peripheral neuropathy [41] and some studies also report associations with severe CTS [39]. SIRI is associated with cardiovascular mortality, and it is a predictive marker for adverse outcomes in children with new-onset T1DM [42]. MLR serves as a predictor of cardiovascular mortality in the general population [43], and a high MLR indicates increased chronic inflammation and can predict certain diabetic complications, such as diabetic nephropathy [44]. PLR has been shown to be helpful in diagnosing several chronic inflammatory diseases, including diabetes mellitus [45].

AISI was reported to be positively associated with the risk of all-cause mortality in sepsis-related acute kidney injury [46]. And also considered by some studies as a prognostic biomarker in idiopathic pulmonary fibrosis [47]. SIII was linked to an increased risk of major adverse cardiovascular events and death in T2DM patients [48]. None of these systemic inflammatory markers correlated with moderate-to-severe CTS in our study.

TyG is a surrogate marker used for the evaluation of insulin resistance in some studies [49], and was shown to be associated with an increased risk of CTS [50]. A higher TyG, reflecting an impaired glucose–lipid homeostasis, was significantly associated with the presence of CTS in our group. TyG has also been shown to predict future macrovascular disease, independently of known cardiovascular risk factors [51]. Another study conducted in China that included 157 patients with T2DM showed that the TyG index correlates with the presence of mild cognitive impairment, with a TyG threshold of 9.45 (AUC = 0.79), sensitivity of 69%, and specificity of 80% [52]. The role of TyG in the progression of microvascular complications in diabetes is unclear, but reports show that an elevated TyG index predicts both their onset and progression, with this association being more evident in diabetic nephropathy and retinopathy [53]. Therefore, we could speculate, given the results of our study, that higher insulin resistance (indicated by elevated TyG) would also predispose to the occurrence of carpal tunnel compression neuropathy in patients with T2DM.

The results support the hypothesis that the severity of CTS in patients with T2DM is influenced by both metabolic imbalance and systemic inflammatory status. Persistent hyperglycemia induces oxidative stress, favoring the accumulation of advanced glycation end products (AGEs), which, through the interaction with the AGE-specific receptors, induce functional and structural neuronal damage [54,55]. AGEs drive microangiopathic alterations in the vasa nervorum, reducing neural perfusion and increasing peripheral nerve vulnerability to chronic metabolic and ischemic damage [54]. The elevated HbA1c and glycemia values in our study suggest that hyperglycemia contributes to median nerve damage. Persistent insulin resistance subsequently promotes metabolic dysfunction through altered glucose utilization, compensatory hyperinsulinemia, adipose tissue lipolysis, chronic low-grade inflammation, ectopic fat deposition, and dysregulated lipid metabolism [49,55]. The significant association of the TyG index with moderate-to-severe forms of CTS in our study suggests a possible role for insulin resistance and metabolic syndrome. In addition, the increase in monocyte levels supports the presence of a chronic systemic inflammatory process, which may contribute to remodeling and fibrosis of the structures within the carpal tunnel.

Although no direct molecular biomarkers were evaluated, the observed associations among HbA1c, the TyG index, monocyte count, and CTS severity suggest potential mechanistic links involving metabolic dysregulation, insulin resistance, chronic low-grade inflammation, oxidative stress, and microvascular nerve injury. These findings may enhance understanding of the pathophysiological substrate underlying median nerve vulnerability in T2DM.

### 3.3. Influence of Demographic, Metabolic, and Occupational Factors on the Occurrence and Severity of CTS in Patients with T2DM

The manifestations of CTS in patients with T2DM may vary between men and women [56]. Generally, in men, electrophysiological changes are more severe, as is diabetic neuropathy, which appears earlier and progresses more rapidly in men, according to some studies [26,56]. In skin biopsies from the wrist, histological studies have shown that men have a lower nerve fiber density, indicating a reduced reserve capacity and greater susceptibility to compressive trauma [16,57]. However, this does not necessarily reflect symptom severity, as women with CTS and diabetic neuropathy often experience more intense symptoms than men [16,57].

Makepeace et al. (2008) conducted a longitudinal observational study in a community of 120,097 people with T2DM that showed a similar incidence of CTS requiring surgical decompression in men and women (*p* = 0.74) [58].

Some data report a prevalence of CTS in the general population of 2.1% in men and 3.0% in women [14]. A systematic review and meta-analysis that included 10 articles published between 2002 and 2017, with a total of 446 diabetic and 2423 non-diabetic patients treated with surgical decompression for carpal tunnel syndrome, reported a mean age of 57.8 years in the diabetic group and 54.8 years in the non-diabetic group. Most patients in both groups were women, accounting for 63% of the diabetic group and 71% of the non-diabetic group [59].

In a study conducted by Gazioglu et al. (2011), no significant differences in age or gender were found in the occurrence of CTS among patients with diabetes, with or without diabetic polyneuropathy, or in a control group [60].

A 2015 meta-analysis that included patients with T1DM and T2DM suggests a modest association between DM and the risk of CTS, with no significant differences between T1DM and T2DM [29]. The reported association between DM and CTS may be confounded. The studies included in this meta-analysis by Pourmemari and Shiri adjusted estimates for age and sex, and the association does not seem to be affected by these variables [29].

An interesting finding from our analysis was an association between female sex and a lower likelihood of moderate-to-severe CTS, which may reflect specific characteristics of the study group, occupational distribution, or the relatively small sample size. The gender distribution is noteworthy because it differs from that of the overall population, where CTS is more commonly reported among women. In the context of diabetes mellitus, this variation might reflect differences in professional activities, metabolic factors, or the severity of diabetic neuropathy.

Mi and Liu (2021) examined the associations of obesity (waist circumference, arm fat mass, arm fat-free mass, BMI) and lipids (HDL-C, LDL-C, total cholesterol, triglycerides) with the risk of CTS [30]. Their findings indicated that adjusted BMI was associated with an increased risk of CTS, whereas dyslipidemia was not. They also found a significant association of CTS with HbA1c (OR = 2.05; 95% CI: 1.01–4.15; *p* = 0.046), but not with fasting insulin or fasting glucose [30].

According to Gazioglu et al. (2011), the average duration of diabetes and metabolic control (HbA1c) were similar between the group with diabetic polyneuropathy without CTS and the group with diabetic polyneuropathy and CTS [60].

Our study examined the predictive value of metabolic and anthropometric factors for carpal tunnel syndrome, emphasizing HbA1c as a significant independent predictor. The finding that elevated HbA1c levels are associated with increased CTS risk supports prior research indicating a link between impaired glucose metabolism and peripheral nerve compression syndromes. Although sex and BMI did not reach statistical significance, their near-threshold *p*-values indicate a possible role that may become clearer in larger samples or more homogeneous populations.

The logistic regression model showed moderate predictive accuracy with an AUC of 0.706. Although not highly discriminative, this performance remains clinically meaningful, especially when early screening and identifying at-risk patients are priorities. The model achieved a sensitivity of 79.2% at the optimal cutoff of 0.356, which might be preferred in clinical settings where reducing false negatives (i.e., missed CTS diagnoses) is an essential goal.

These results suggest that adding HbA1c to routine screening protocols for CTS—especially in patients with suspected metabolic syndrome or diabetes—could improve early detection and intervention. However, due to the model’s moderate specificity and overall accuracy (~63.3%), further refinement and external validation are necessary before clinical use. Future research should include larger sample sizes, explore additional biomarkers, and evaluate the model’s performance in prospective cohorts.

Shiri et al. (2011) conducted a cross-sectional study of the Finnish population, including 6254 subjects, between 2000 and 2001 [2]. They found associations between CTS and cardiovascular risk factors, including obesity, high LDL-C, high triglycerides, high blood pressure, and cardiac arrhythmias, among people aged 30–44 [2]. In those over 60, CTS was linked to coronary artery disease, valvular diseases, and increased intima-media thickness [2].

Habib et al. (2023) conducted a cross-sectional study that included 517 hands from 377 patients with CTS, with and without diabetes mellitus, and found no correlation between CTS severity and duration of diabetes mellitus (F = 0.779; *p* = 0.540) [61].

In our study, comparing clinical–demographic characteristics by CTS severity showed that most evaluated parameters, including age, duration of T2DM, blood pressure, and type of antidiabetic treatment, did not differ significantly between groups. This finding suggests that, within the studied population, the severity of CTS is not directly influenced by these factors.

In contrast, active professional status was strongly associated with moderate/severe CTS, with professionally active patients facing a 3.5-fold higher risk of moderate/severe CTS. This supports the idea that occupational factors, possibly related to repetitive hand motions and mechanical stress on the radiocarpal joint and median nerve, may contribute to the development of compressive neuropathy. Our study data suggests that ongoing professional activity is a risk factor for more severe median compressive neuropathy, even among those with T2DM. The high number of retired patients primarily reflects the study group’s age distribution, with a median age of about 69 years.

### 3.4. Electrophysiological Studies

Electrophysiological studies are often used to diagnose compression neuropathies, but approaches can vary by country. Some studies indicate that about 70% of patients with CTS have had preoperative electrophysiological tests, which are especially helpful for differential diagnosis and estimating preoperative prognosis [16,62]. In patients with diabetes, electrophysiological testing of the lower limbs is also useful for evaluating the presence and severity of diabetic neuropathy [16].

A study conducted in India examined the relationships between NCS parameters and various variables among patients with T2DM. It highlighted a significant negative correlation (*p* < 0.05) between disease duration and all analyzed NCS parameters. Additionally, it found a negative correlation between fasting blood glucose and both motor conduction velocity and SNAP amplitude. The study also observed a negative correlation between postprandial blood glucose levels and motor conduction velocity, sensory conduction velocity, and SNAP amplitude [63].

Some studies have shown that postoperative electrophysiological parameters in patients with CTS improve over five years, and the results are not affected by the presence of diabetic neuropathy [16,64]. Other studies show that concomitant diabetic neuropathy is linked to more residual symptoms [16], and that patients with concomitant diabetic retinopathy recover more slowly than those with T2DM without retinopathy [65].

Kim et al. (2017) reported that the motor conduction velocity of the right median nerve was lower and the SNAP latency was longer in patients with DM and CTS than in those without diabetes but with CTS [9]. However, after performing multivariate analysis, it was shown that only the SNAP amplitude at the median nerve level in women was the only variable significantly associated with the presence of diabetes mellitus (*p* < 0.001) [9].

The results of studies on electrophysiological parameters in patients with diabetes and CTS are inconsistent. Thus, Tony et al. (2018) found significant differences between the CTS group without diabetes and the CTS group with diabetes in median nerve CMAP amplitude and median nerve SNAP latency, but no significant differences in distal motor latencies [66].

The study by Park et al. (2013), which evaluated the electrophysiological characteristics of patients with carpal tunnel syndrome, with and without diabetes, showed that SNAP abnormalities in patients with mild carpal tunnel syndrome were more pronounced in the group with diabetes [7]. Meanwhile, in moderate to severe carpal tunnel cases, the CMAP and SNAP amplitudes were more affected in the diabetic group, suggesting a polyneuropathic type of damage associated with diabetes [7,67].

In a study involving 105 CTS patients, 22 of whom had DM, Kim et al. (2017) found no significant differences between CMAP and SNAP in patients with and without diabetes mellitus [9]. This suggests that thin, unmyelinated fibers are most vulnerable to diabetes-induced microvascular changes [68], whereas electrophysiological testing primarily reflects changes in myelinated nerve fibers [12].

Gazioglu et al. (2011) demonstrated in their study that distal motor and sensory latencies of the median nerve were significantly prolonged in both the group of patients with DM and CTS with diabetic peripheral neuropathy and the group of patients with DM without CTS but with peripheral neuropathy, compared to the control group [60].

Prolonged distal median nerve latency is more common in patients with diabetic polyneuropathy [60] and these results are similar to our findings.

In our study, the correlation analysis indicates a significant association between lower-limb peripheral nerve damage and median nerve dysfunction at the carpal tunnel level. The positive correlations observed indicate that higher amplitudes of the tibial and sural nerves are associated with faster median motor and sensory conduction velocities, suggesting that neurophysiological damage to the median nerve may, at least in part, reflect the severity of systemic diabetic peripheral neuropathy.

The fact that the strongest associations were found for the sural and tibial nerves supports the presence of a length-dependent distal sensorimotor neuropathy typical of T2DM. These findings suggest that in patients with T2DM, CTS should not be seen solely as a local compressive neuropathy, but also as part of a broader issue involving increased vulnerability of peripheral nerves due to metabolic and microvascular factors.

Other studies have combined electrophysiological investigations with ultrasound measurements, such as median nerve cross-sectional area (CSA), and the results have been inconsistent. Kotb et al. (2018) reported a significant difference in median nerve CSA in diabetes-associated CTS [69], whereas other studies did not show significant differences between patients with and without DM with CTS [9].

In the study by Hussain et al. (2014), a trend toward lower ulnar motor nerve conduction velocity and median sensory nerve conduction velocity was observed in patients with diabetic neuropathy and a shorter duration of diabetes than in those without neuropathy, although the differences were not statistically significant [70]. Conversely, these values are significantly lower in patients with a longer duration of diabetes and peripheral neuropathy, a finding partially confirmed in our study [70]. Nerve conduction velocity decreases with increasing severity of neuropathy and with increasing duration of T2DM, and these changes are similar for both motor and sensory nerves [69], partially mirroring our study’s findings.

Hussain et al. (2014) showed that conduction velocity in the sural nerve decreases after a shorter duration of diabetes mellitus, whereas sensory conduction velocity in the median nerve decreases in the upper limbs, though not statistically significant [70]. In patients with a longer duration of diabetes mellitus, the sensory conduction velocity decreases in both the lower and upper limbs, confirming the pattern of length-dependent neuropathy [70].

The results of the correlation matrix support the hypothesis that electroneurographic changes in the median nerve of patients with T2DM and CTS are associated with generalized peripheral nerve damage. The significant correlations observed between the median parameters and those of the ulnar, tibial, and sural nerves suggest that median nerve dysfunction does not exclusively reflect a local compressive mechanism but rather a broader context of systemic peripheral neuropathy.

The strong association with sural nerve parameters is particularly important, as the sural nerve is a classic marker of distal sensory diabetic neuropathy. This supports the idea that the severity of CTS may be increased by metabolically induced nerve vulnerability, endoneural microangiopathy, and length-dependent axonal degeneration. In addition, the correlations with the tibial and peroneal motor nerves suggest that the damage extends to motor fibers, reinforcing the hypothesis of a generalized sensorimotor neuropathy associated with T2DM.

To our knowledge, this is the first study in our geographical area to examine patients with T2DM using NCS and to assess the association between T2DM and CTS.

The novelty of the present study lies in its integrated evaluation of clinical, metabolic, inflammatory, and electrophysiological determinants of CTS severity in patients with T2DM. Unlike previous studies that focused mainly on CTS prevalence or isolated metabolic risk factors, our study simultaneously assessed glycemic control, insulin resistance (as evaluated by the TyG index), systemic inflammatory parameters, and nerve conduction abnormalities in both upper and lower limbs. In particular, the association between monocyte count and CTS severity, together with the relationship between median nerve dysfunction and abnormalities of the tibial and sural nerves, supports the hypothesis that CTS severity in T2DM may reflect not only focal compression neuropathy but also broader diabetic peripheral nerve vulnerability. Furthermore, the evaluation of the TyG index in relation to CTS severity remains underexplored in the current literature.

### 3.5. Limitations

Our study has several limitations. Firstly, it is a single-center study, so the number of patients included is small, which may have decreased the statistical power. Based on the sample size, we calculated a Cohen’s d of 0.51, meaning this sample size can detect a moderate effect size. The study included only Caucasian patients, limiting the generalizability of the results.

A limitation of the present study is the absence of a control group comprising subjects without DM. However, the primary aim was not the comparison of CTS prevalence between individuals with and without DM, but rather to identify metabolic, inflammatory, and electrophysiological factors associated with CTS severity within a homogeneous T2DM cohort. The study was designed as a within-cohort analysis of patients with T2DM, in which participants were classified by electrophysiological CTS severity and compared between the resulting groups (moderate/severe CTS versus absent/mild CTS). This approach allowed us to investigate determinants of clinically relevant CTS severity within the T2DM population, rather than differences between individuals with or without DM.

The study population may not be fully representative of the general population with T2DM, since participants were recruited from a tertiary care setting and underwent systematic electrophysiological evaluation. This may have increased the detection of mild or subclinical forms of CTS and contributed to the relatively high prevalence observed. Therefore, caution is required when extrapolating prevalence estimates to broader populations with T2DM.

Another limitation of the study is that cases with diabetic polyneuropathy were not excluded, which could influence CMAP and SNAP amplitudes at the median nerve level, as well as, to a lesser extent, latency and nerve conduction velocity, but it also revealed the association between the severity of CTS and increased vulnerability of peripheral nerves.

## 4. Materials and Methods

### 4.1. Study Design, Patient Selection, and Eligibility Criteria

This cross-sectional study was conducted from June 2023 to June 2024 and was approved by the Scientific Research Ethics Committee of the “George Emil Palade” University of Medicine, Pharmacy, Science and Technology of Târgu Mureș, Romania (approval no. 2396/14 June 2023).

The patients included in the study were referred from the Neurology and Diabetology Outpatient clinics of the Emergency County Hospital of Târgu Mureș or were recruited from patients admitted to the Neurology Clinic for evaluation, all of whom originated from the public healthcare system. The patients were consecutively included in the study, and all of them signed an informed consent at study entry. This study was conducted in accordance with the principles of the Declaration of Helsinki.

The study included adults aged 18 years or older diagnosed with T2DM. The diagnosis of diabetes mellitus was confirmed by a diabetologist according to the American Diabetes Association guidelines: fasting plasma glucose ≥ 126 mg/dL, 2 h plasma glucose ≥ 200 mg/dL during a 75 g oral glucose tolerance test, glycosylated hemoglobin (HbA1c) ≥ 6.5%, or random plasma glucose ≥ 200 mg/dL [71]. The duration of diabetes was determined through patient interviews or medical records.

Exclusion criteria: patients with pregnancy, history of endocrinopathies (such as thyroid disease) or other underlying disorders associated with CTS (like renal and hepatic diseases), other types of diabetes, previous median nerve trauma, rheumatoid arthritis, prior wrist surgery, or conditions that mimic CTS (such as cervical radiculopathy or brachial plexopathy), neuromuscular junction disorders, muscle diseases, or chronic alcohol use, exposure to exogenous toxins or metals, and skin lesions or edema that could interfere with NCS.

General information about patients, such as age, sex, nationality, marital status, income, and smoking history, was obtained through questionnaires and direct interviews, which were completed on the same day as the NCS. The height, weight, and waist circumference were obtained by standard methods, and the BMI was calculated using the formula: [weight (kg)/height (m)^2^] [72]. We divided the patients into 4 age groups: under 60, 60–65, 66–70, and over 70. These age groups were used in the multivariate binary logistic regression.

On the day of clinical evaluation, blood was drawn in a fasting condition for subsequent analyses of several parameters: blood glucose, HbA1c, total cholesterol, LDL-C, HDL-C, triglycerides, uric acid, serum albumin, blood urea nitrogen, creatinine, eGFR, and fibrinogen. The TyG index was used as a biomarker of insulin resistance and calculated using the formula [73]:

TyG = ln (fasting triglycerides [mg/dL] × fasting glucose [mg/dL])/2

Several systemic inflammatory markers were calculated from the complete blood count (CBC) data. The NLR was calculated as the absolute neutrophil count divided by the absolute lymphocyte count [74]. The MLR was calculated as the absolute monocyte count divided by the lymphocyte count [43], while PLR was calculated as the absolute platelet count divided by the lymphocyte count [45]. The SIRI, a new marker of microinflammation, was calculated as the product of neutrophil and monocyte counts divided by the lymphocyte count [42]. AISI is the product of the absolute number of neutrophils, monocytes, and platelets, divided by lymphocytes [75]. The SIII is calculated by multiplying the absolute platelet count by the absolute neutrophil count, then dividing that product by the absolute peripheral lymphocyte count [75].

### 4.2. Nerve Conduction Studies Protocol

Electrophysiological assessment of the upper and lower limbs was performed for all patients according to the recommended American Association of Electrodiagnostic Medicine protocol [76] using a four-channel EMG apparatus (Nihon Kohden, Neuropack MEB-9400, Tokyo, Japan) in the Neurophysiological Unit. Conduction velocities were measured using standard stimulation methods and recorded with surface electrodes coated with conductive gel and secured with adhesive tape [77,78]. For each patient, bilateral recordings were obtained from the median, ulnar (sensory and motor), common peroneal, tibial, and sural nerves. All tests were conducted with the patients in the supine position. The temperature in the examination room was maintained at 23–25 °C [79].

A bipolar surface electrode, with the anode positioned 2.5 cm proximal to the cathode, was used for nerve stimulation. The antidromic method was employed to record SNAPs. Onset latency was measured to determine latency, and baseline-to-peak was used to assess amplitude [59]. The sensory conduction velocity (m/s) was calculated based on the latency and the distance between the stimulating and recording electrodes [78].

CMAPs were recorded using the belly tendon method, and motor nerve conduction velocity was determined by stimulating a peripheral nerve and recording the response from the belly of a muscle innervated by that nerve. The device recorded the latency of the motor response, which indicates the time it takes for the electrical impulse to travel from the stimulation site to the recording site, expressed in milliseconds. The nerve conduction velocity (m/s) was calculated by stimulating two distinct points along the same nerve, measuring the distance between them, and recording the onset latencies of the motor response [78].

The median motor nerve was stimulated at the wrist and antecubital fossa, with CMAP recorded from the abductor pollicis brevis muscle. Onset distal latency, conduction velocity, and distal CMAP amplitude were documented. For the sensitivity study, the antidromic method was employed using wrist stimulation, with SNAP recorded from finger 1. The sensitivity studies assessed SNAP amplitude, onset latency, and conduction velocity. A latency difference between the median and ulnar nerves of ≥0.5 msec was considered abnormal and was recorded from finger 4 [80].

Motor and sensory conduction velocities, CMAP and SNAP amplitudes at distal stimulation, and onset latency at distal stimulation were recorded.

### 4.3. Electrophysiological Diagnosis, Severity Classification, and Statistical Grouping of Carpal Tunnel Syndrome

The electrophysiological diagnosis of CTS was confirmed by NCS based on the following criteria: increased latency to distal stimulation of the median sensory and motor nerves, a median-ulnar sensory latency difference of over 0.5 msec on ring-finger studies, and reduced conduction velocities in the median sensory and motor nerves. Additionally, CMAP and SNAP amplitudes were measured to assess secondary axonal loss. The laboratory reference parameters used to evaluate the median nerve included: distal sensory latency < 3.4 ms, SNAP amplitude > 20 μV, distal motor latency < 4 ms, CMAP amplitude > 5.4 mV, and motor and sensory nerve conduction speed < 50 m/s.

CTS severity was categorized using a laboratory-adapted electrophysiological classification based on the extent of sensory and motor nerve involvement, inspired by previously published neurophysiological grading systems, particularly those proposed by Padua et al. (1997) and Bland et al. (2000) [81,82]. Mild CTS was defined by isolated sensory abnormalities, including prolonged distal sensory latency and/or a median–ulnar sensory latency difference > 0.5 ms, with preserved motor conduction and amplitudes. Moderate CTS was characterized by sensory abnormalities with motor fiber involvement, as reflected by prolonged distal motor latency and/or reduced sensory amplitude, without clear evidence of motor axonal loss. Severe CTS was characterized by marked electrophysiological impairment, including combined sensory and motor abnormalities, absent sensory responses, or reduced CMAP amplitudes suggestive of secondary axonal damage [81,82]. To standardize severity grading within the study cohort, deviations exceeding 20% from local laboratory reference values were considered indicative of clinically relevant electrophysiological impairment.

### 4.4. Statistical Analyses

First, the patients were divided into two groups: one with patients who met the electrophysiological criteria for diagnosing CTS, and another with patients who had normal nerve conduction study values. Patients were initially classified into three levels of carpal tunnel syndrome severity: mild, moderate, and severe.

For the statistical analysis, patients were later divided into two groups: the “normal + mild damage” group and the “moderate + severe damage” group. This approach was chosen to minimize the risk of overdiagnosing compressive neuropathy in patients with diabetes mellitus, since in this population it can often be linked to distal symmetric diabetic polyneuropathy. Diffuse peripheral neuropathy can affect median nerve electrophysiological parameters, particularly latencies and sensory amplitudes, potentially leading to overestimation of mild CTS [16]. Thus, grouping normal and mildly affected cases together allowed for a clearer distinction between minor changes—possibly due to diabetic neuropathy—and those with clear clinical and electrophysiological significance, represented by moderate and severe impairment.

The data were collected and organized using Microsoft Excel 365 (Microsoft Corporation, Redmond, WA, USA). IBM SPSS Statistics for Windows, version 31.0 (IBM Corp., Armonk, NY, USA), was used for the statistical analysis. Normality of continuous variables was assessed using the Shapiro–Wilk normality test. Continuous variables were summarized as mean ± SD for Gaussian distributions and as median (IQR) for non-Gaussian distributions. Qualitative variables were summarized as frequencies and percentages. Pearson correlation test was used for numerical data with a Gaussian distribution, and Spearman correlation test was used for data without a Gaussian distribution.

Comparisons between groups were performed using Pearson’s chi-square test or Fisher’s exact/Fisher-Freeman-Halton test, as appropriate, for qualitative variables. For continuous variables with a normal distribution, Student’s *t*-test (for two independent groups) or analysis of variance (ANOVA) (for three or more independent groups) was used to compare central tendencies. For non-normal distributions, the Mann–Whitney U test (for two independent groups) or the Kruskal–Wallis test (for three or more independent groups) was used. A multivariable binary logistic regression model was used to assess the diagnosis of CTS. Sex, BMI, and HbA1c were selected as predictor variables due to their clinical significance, unique contribution, and the limited number of CTS-positive cases (*n* = 54). Sex served as a demographic control, BMI as a known risk factor for metabolic and anthropometric outcomes, and HbA1c as an indicator of long-term glycemic control. Blood glucose levels and the TyG index were excluded from the main multivariable model to avoid redundancy with HbA1c and BMI, respectively, and to minimize multicollinearity and overfitting. MLR and NLR were also evaluated using univariable binary logistic regression models; however, neither showed a statistically significant association with the presence of CTS. An ROC analysis was applied to the binary logistic regression model to determine the AUC and the optimal cutoff value using the Youden index. A *p*-value < 0.05 was considered statistically significant across all tests.

A post hoc analysis examined the sample size and statistical power for the main comparison between patients with absent/mild CTS and those with moderate/severe CTS. The final group included 123 patients: 69 with absent or mild CTS and 54 with moderate or severe CTS. Using an alpha of 0.05, 80% power, and the observed group ratio, we obtained a Cohen’s d of 0.51, indicating this sample can detect a moderate effect size for continuous outcomes. The study is sufficiently powered to reveal moderate differences between groups but may not detect smaller effects.

## 5. Conclusions

The results support the significant role of metabolic imbalance and poor glycemic control in the development and progression of median nerve damage. Elevated HbA1c, admission glucose levels, and TyG index values were significantly associated with moderate and severe cases, with HbA1c emerging as an independent predictor of CTS severity, underscoring the impact of chronic hyperglycemia on nerve vulnerability.

Furthermore, the association with elevated monocyte counts suggests a chronic systemic inflammatory condition that, together with microvascular and metabolic factors, may promote endoneural damage and structural changes within the carpal tunnel.

A key aspect of the study is demonstrating the link between median nerve damage and electrophysiological changes in the peripheral nerves of the lower limbs. The notable correlations with the sural, tibial, and peroneal nerves support the idea that, in patients with T2DM, CTS is not just a localized compressive neuropathy but part of a broader pattern of generalized diabetic peripheral neuropathy and an increased systemic vulnerability of nerve fibers to metabolic and mechanical stress.

Additionally, professional activity was linked to a significantly higher risk of moderate and severe forms, indicating the extra impact of occupational factors and repetitive microtrauma.

Overall, our data support the need for an integrated metabolic, inflammatory, and neurophysiological approach when assessing patients with T2DM and symptoms suggestive of CTS. They also highlight the value of early electrophysiological screening to identify those at increased risk of neurological progression.

## Figures and Tables

**Table 1:** Comparison of group characteristics between the absent/mild CTS (−) group and the moderate/severe CTS (+) group.

Variable	All Patients (*n* = 123)	CTS (−) Patients (*n* = 69)	CTS (+) Patients (*n* = 54)	*p*
Age (years)	69.00 (61.00–75.00)	70 (63.5–75)	69.00 (58.75–74.00)	0.315
Age group				
Under 60, *n* (%)	28 (22.8)	12 (17.4)	16 (29.6)	0.423
60–65, *n* (%)	17 (13.8)	11 (15.9)	6 (11.1)	
66–70, *n* (%)	21 (17.1)	12 (17.4)	9 (16.7)	
Over 70, *n* (%)	57 (46.3)	34 (49.3)	23 (42.6)	
Sex				
Female sex, *n* (%)	58 (47.2)	38 (55.1)	20 (37.0)	0.047
Male sex, *n* (%)	65 (52.8)	31 (44.9)	34 (63.0)	
BMI (kg/m^2^)	29.75 (26.67–33.46)	26.69 ± 5.19	31.60 ± 5.55	0.053
BMI over 25 (kg/m^2^) *n* (%)	107 (86.9)	58 (84)	49 (90.7)	0.419
Height (cm)	166.64 ± 9.06	167.35 ± 8.53	165.74 ± 9.72	0.339
Weight (kg)	83.00 (72.00–98.00)	80 (70–99)	86.00 (75.00–96.25)	0.202
Abdominal circumference (cm)	109.51 ± 13.46	107.5 ± 13.9	112.29 ± 12.46	0.065
T2DM duration (years)	12.00 (6.00–20.00)	11 (5–16)	12.00 (6.00–20.00)	0.415
Systolic BP (mmHg)	140.00 (130.00–147.00)	136 (129–144)	140.00 (130.00–150.00)	0.345
Diastolic BP (mmHg)	80.00 (73.00–86.00)	79.62 ± 8.74	79.44 ± 9.11	0.913
Any history of smoking, *n* (%)	51 (41.5)	32 (46.4)	19 (35.2)	0.211
Marital status				
Married *n* (%)	84 (68.3)	45 (65.2)	39 (72.2)	0.388
Single, *n* (%)	7 (5.7)	3 (4.4)	4 (7.5)	
Widowed, *n* (%)	32 (26)	21 (30.4)	11 (20.3)	
Nationality				
Romanian, *n* (%)	98 (79.7)	55 (79.7)	43 (79.6)	0.944
Hungarian, *n* (%)	19 (15.4)	11 (15.9)	8 (14.8)	
Other, *n* (%)	6 (4.9)	3 (4.3)	3 (5.6)	
Employment status				
Actively employed, *n* (%)	25 (20.3)	8 (11.5)	17 (31.4)	0.013
Retired, *n* (%)	98 (79.7)	61 (88.5)	37 (68.6)	
Income				
Under 390 €, *n* (%)	52 (42.3)	26 (37.7)	26 (48.1)	0.482
390–590 €, *n* (%)	65 (52.8)	39 (56.5)	26 (48.1)	
Over 590 €, *n* (%)	6 (4.9)	4 (5.8)	2 (3.7)	
Therapy for T2DM				
Oral Antidiabetic Treatment, *n* (%)	107 (87)	57 (82.6)	50 (92.6)	0.115
Metformin, *n* (%)	99 (80.5)	54 (78.3)	45 (83.3)	0.481
Sulfonylurea, *n* (%)	25 (20.3)	11 (15.9)	14 (25.9)	0.172
DPP4-inhibitors, *n* (%)	14 (11.4)	6 (8.7)	8 (14.8)	0.289
SGLT2-inhibitors, *n* (%)	28 (22.8)	17 (24.6)	11 (20.4)	0.575
AR GLP-1 oral, *n* (%)	1 (0.8)	1 (1.4)	0 (0.0)	1.000
AR GLP-1 injectable, *n* (%)	4 (3.3)	3 (4.3)	1 (1.9)	0.630
Insulin treatment, *n* (%)	48 (39)	24 (34.8)	24 (44.4)	0.276
Both Insulin and Oral treatment, *n* (%)	41 (33.3)	19 (27.5)	22 (40.7)	0.123

Legend: Continuous variables are presented as mean ± SD for normally distributed data, and as median (IQR) for non-normally distributed data. Categorical variables are shown as counts (percentages). CTS (−) = absent/mild; CTS (+) = moderate/severe. Continuous variables with normal distribution were analyzed using Student’s *t*-test, whereas non-normally distributed ones were examined with the Mann–Whitney test. Categorical variables were assessed using Pearson’s chi-square test or Fisher’s exact test. DPP4 = dipeptidyl peptidase-4; SGLT2 = sodium–glucose cotransporter 2; AR GLP-1 = glucagon-like peptide-1 receptor agonists.

**Table 2:** Comparison of biological, metabolic parameters, and inflammatory markers between patients with absent or mild carpal tunnel syndrome (CTS (−)) and those with moderate to severe carpal tunnel syndrome (CTS (+)).

	All Patients (*n* = 123)	CTS (−) Patients (*n* = 69)	CTS (+) Patients (*n* = 54)	*p*
HbA1c (%)	7.08 (6.49–8.30)	6.83 (6.36–7.48)	7.44 (6.66–9.52)	0.004
Blood glucose (mg/dL)	135.00 (111.00–166.00)	120.00 (101.00–154.50)	150.00 (125.50–188.00)	<0.001
Uric acid (mg/dL)	5.70 (4.31–6.63)	5.60 (4.32–6.67)	5.73 (4.30–6.45)	0.809
Total Cholesterol (mg/dL)	165.00 (135.00–200.00)	169.00 (141.00–198.50)	158.50 (118.50–206.75)	0.347
LDL-Cholesterol (mg/dL)	92.00 (65.70–121.50)	99.60 (74.05–124.50)	86.60 (58.05–116.50)	0.119
HDL-Cholesterol (mg/dL)	45.00 (37.08–53.70)	45.40 (36.60–56.55)	43.20 (38.35–50.10)	0.37
Triglycerides (mg/dL)	135.00 (96.95–209.50)	128.00 (95.95–195.00)	151.50 (97.50–250.00)	0.337
TyG	9.2 ± 0.7	9.06 ± 0.63	9.37 ± 0.74	0.018
Blood urea nitrogen (mg/dL)	38.52 (30.90–49.22)	38.52 (31.85–46.80)	38.52 (29.87–51.57)	0.831
Serum creatinine (mg%)	0.82 (0.67–1.06)	0.8 (0.68–1.03)	0.86 (0.7–1.06)	0.352
eGFR (mL/min/1.73 m^2^)	89.52 (72.30–104.60)	91.08 ± 26.26	89.81 ± 26.67	0.792
Serum albumin (g/dL)	4.4 ± 0.35	4.37 ± 0.34	4.44 ± 0.36	0.316
Serum fibrinogen (mg/dL)	341.03 (285.12–393.58)	345.48 (296.00–400.00)	332.36 (276.36–375.13)	0.277
Albumin/Fibrinogen Ratio	76.31 (62.03–90.32)	80.78 (63.70–94.53)	72.39 (60.55–89.02)	0.194
Platelet number (10^3^/uL)	233.00 (201.00–284.00)	233.00 (201.50–293.00)	236.00 (197.25–279.00)	0.46
Monocytes	0.64 (0.51–0.75)	0.56 (0.46–0.73)	0.7 (0.54–0.8)	0.012
Neutrophil-to-lymphocyte ratio (NLR)	2.43 (1.75–3.21)	2.48 (1.80–3.25)	2.36 (1.74–3.16)	0.37
Systemic Immune-Inflammation Index (SIII)	565.38 (401.74–828.66)	598.37 (449.46–834.91)	496.53 (381.19–810.48)	0.161
Monocyte-to-lymphocyte ratio (MLR)	0.30 (0.23–0.37)	0.30 (0.23–0.37)	0.30 (0.24–0.39)	0.706
PLR	118.48 (86.16–142.35)	126.95 (94.26–147.72)	105.96 (81.22–128.67)	0.009
Systemic Inflammation Response Index (SIRI)	1.53 (1.14–2.02)	1.45 (1.15–1.92)	1.70 (1.12–2.38)	0.498
Aggregate Index of Systemic Inflammation (AISI)	355.54 (238.31–536.09)	353.67 (245.44–535.93)	360.21 (237.38–560.29)	0.887

Legend: Continuous variables are presented as mean ± SD for normally distributed data, and as median (IQR) for non-normally distributed data. Categorical variables are shown as counts (percentages). CTS (−) = absent/mild; CTS (+) = moderate/severe. Continuous variables with normal distribution were analyzed using Student’s *t*-test, whereas non-normally distributed ones were examined with the Mann–Whitney test. LDL = low-density lipoprotein; HDL = high-density lipoprotein; TyG = Triglyceride–Glucose Index; eGFR = estimated glomerular filtration rate; PLR = platelet-to-lymphocyte ratio.

**Table 3:** Multivariable binary logistic regression models for predictors of moderate-to-severe carpal tunnel syndrome.

Predictor	OR	95% CI	*p*-Value	Bootstrap *p*-Value
Model 1: sex, BMI, and HbA1c				
Sex	2.09	0.944–4.629	0.057	0.07
BMI	1.071	0.996–1.151	0.076	0.059
HbA1c	1.6	1.199–2.134	0.001	0.002
Model 2: age-adjusted model				
Age category, overall	—	—	0.45	—
60–65 years old	0.341	0.083–1.405	0.136	0.14
66–70 years old	0.706	0.198–2.510	0.59	0.628
>70 years old	0.911	0.310–2.681	0.866	0.876
Sex	2.194	0.954–5.047	0.065	0.058
BMI	1.07	0.991–1.156	0.083	0.084
HbA1c	1.688	1.239–2.300	<0.001	<0.001

Legend: OR = odds ratio; CI = confidence interval; BMI = body mass index; Model 1 included sex, BMI, and HbA1c. Model 2 additionally included age category, with patients aged <60 years as the reference group. Bootstrap results were based on 1000 samples.

**Table 4:** Electroneurographic Parameters of the Peripheral Nerves in the Lower Limbs Related to Carpal Tunnel Syndrome Severity.

	Carpal Tunnel Syndrome		*p*
	CTS (−) (*n* = 69)	CTS (+) (*n* = 54)	
Left common peroneal nerve ankle amplitude (mV)	3.2 (2.3–4.05)	2.35 (1.425–3.85)	0.074
Left tibial nerve ankle amplitude (mV)	5.3 (2.65–6.45)	4.5 (2.475–5.5)	0.028
Left sural nerve amplitude (μV)	10.9 (7.05–13.7)	7.6 (6.5–10.875)	0.004
Right common peroneal nerve ankle amplitude (mV)	3.3 (2.25–4.05)	2.8 (1.6–3.6)	0.088
Right tibial nerve ankle amplitude (mV)	5.4 (2.35–6.65)	4.6 (2.55–6.225)	0.259
Right sural nerve amplitude (μV)	11 (7.5–14.65)	7.65 (6.775–10.85)	0.014

Legend: Data are shown as median (IQR). CTS (−) = absent/mild; CTS (+) = moderate/severe. Continuous variables with normal distribution were analyzed using Student’s *t*-test, whereas non-normally distributed ones were examined with the Mann–Whitney test.

**Table 5:** Correlations between motor and sensory conduction velocities of the median nerve and CMAP and SNAP amplitudes of the peripheral nerves of the lower limbs.

	Left Motor Median Nerve Conduction Speed (m/s) (r, *p*)	Right Motor Median Nerve Conduction Speed (m/s) (r, *p*)	Left Median Nerve Sensory Conduction Speed (m/s) r, *p*	Right Median Nerve Sensory Conduction Speed (m/s) r, *p*
Left common peroneal nerve ankle amplitude (mV)	0.219, 0.015	0.234, 0.009	0.209, 0.021	0.115, 0.204
Right common peroneal nerve ankle amplitude (mV)	0.228, 0.011	0.291, 0.001	0.177, 0.05	0.046, 0.615
Left tibial nerve ankle amplitude (mV)	0.378, <0.001	0.305, 0.001	0.206, 0.022	0.226, 0.012
Right tibial nerve ankle amplitude (mV)	0.313, <0.001	0.293, 0.001	0.159, 0.078	0.081, 0.373
Left sural nerve amplitude (uV)	0.369, <0.001	0.352, <0.001	0.182, 0.044	0.194, 0.032
Right sural nerve amplitude (uV)	0.348, <0.001	0.349, <0.001	0.15, 0.097	0.195, 0.031

Legend: Values are expressed as Spearman’s correlation coefficient (r) and *p*-value. Positive correlations suggest that higher amplitudes of lower-limb peripheral nerve potentials are associated with faster median nerve motor and sensory conduction velocities.

**Table 6:** Spearman correlation matrix between latency, amplitude, and conduction velocity of the median nerve and the ulnar, common peroneal, tibial, and sural nerves.

		Median Motor (r, *p*)			Median Sensitive (r, *p*)		
		Latency	A. CMAP	Speed	Latency	A. CMAP	Speed
Median nerve	Latency	-	−0.467, <0.001	−0.388, <0.001	0.634, <0.001	−0.601, <0.001	−0.601, <0.001
	A. CMAP	−0.467, <0.001	-	0.244, <0.001	−0.248, <0.001	0.341, <0.001	0.302, <0.001
	Speed	−0.388, <0.001	0.244, <0.001	-	−0.300, <0.001	0.500, <0.001	0.327, <0.001
Ulnar nerve	Latency	0.306, <0.001	−0.041, 0.522	−0.309, <0.001	0.357, <0.001	−0.244, <0.001	−0.243, <0.001
	A. CMAP	−0.009, 0.887	0.180, 0.005	0.112, 0.08	−0.013, 0.841	0.176, 0.006	0.031, 0.627
	Speed	−0.142, 0.026	0.045, 0.484	0.413, <0.001	−0.207, 0.001	0.259, <0.001	0.205, 0.001
Common peroneal nerve	Latency	0.062, 0.331	−0.008, 0.902	−0.205, 0.001	0.121, 0.058	−0.141, 0.028	−0.063, 0.322
	A. CMAP	−0.114, 0.076	0.172, 0.007	0.255, <0.001	−0.083, 0.194	0.232, <0.001	0.127, 0.047
	Speed	−0.229, <0.001	0.071, 0.268	0.444, <0.001	−0.323, <0.001	0.404, <0.001	0.269, <0.001
Tibial nerve	Latency	0.137, 0.031	−0.02, 0.751	−0.062, 0.33	0.049, 0.444	−0.02, 0.756	−0.033, 0.608
	A. CMAP	−0.143, 0.024	0.205, 0.001	0.338, <0.001	−0.122, 0.055	0.332, <0.001	0.135, 0.034
	Speed	−0.223, <0.001	0.102, 0.11	0.491, <0.001	−0.255, <0.001	0.381, <0.001	0.240, <0.001
Median sensitive	Latency	0.634, <0.001	−0.248, <0.001	−0.300, <0.001	-	−0.475, <0.001	−0.870, <0.001
	A. SNAP	−0.601, <0.001	0.341, <0.001	0.500, <0.001	−0.475, <0.001	-	0.500, <0.001
	Speed	−0.601, <0.001	0.302, <0.001	0.327, <0.001	−0.870, <0.001	0.500, <0.001	-
Ulnar sensitve	Latency	0.11, 0.084	0.038, 0.55	−0.206, 0.001	0.153, 0.016	−0.127, 0.047	−0.052, 0.42
	A. SNAP	−0.135, 0.034	0.093, 0.145	0.236, <0.001	−0.172, 0.007	0.336, <0.001	0.122, 0.057
	Speed	−0.185, 0.004	0.119, 0.063	0.220, 0.001	−0.171, 0.007	0.227, <0.001	0.216, 0.001
Sural	Latency	−0.061, 0.337	−0.014, 0.824	−0.008, 0.902	−0.015, 0.814	0.081, 0.207	0.006, 0.928
	A. SNAP	−0.219, <0.001	0.138, 0.031	0.359, <0.001	−0.202, 0.001	0.462, <0.001	0.181, 0.004
	Speed	−0.045, 0.48	0.06, 0.35	0.127, 0.046	−0.071, 0.269	−0.034, 0.598	0.121, 0.058

Legend: Values are expressed as Spearman’s correlation coefficient (r) and *p*-value.

## Data Availability

The data presented in this study are available on request from the corresponding author. The data is not publicly available due to privacy reasons.

## Supplementary Materials

The following supporting information can be downloaded at https://www.mdpi.com/article/10.3390/ijms27114995/s1.

## Abbreviations

The following abbreviations are used in this manuscript:

AGEs	Advanced glycation end products
AISI	Aggregate Index of Systemic Inflammation
ANOVA	Analysis of variance
AUC	Area under the curve
BMI	Body mass index
BP	Blood pressure
CBC	Complete blood count
CI	Confidence interval
CMAP	Compound muscle action potentials
CTS	Carpal tunnel syndrome
CTS (−)	Absent or mild carpal tunnel syndrome
CTS (+)	Moderate to severe carpal tunnel syndrome
DM	Diabetes mellitus
eGFR	Estimated glomerular filtration rate
EMG	Electromyography
HbA1c	Hemoglobin A1c
HDL-C	High-density lipoprotein cholesterol
IQR	Interquartile range
LDL-C	Low-density lipoprotein cholesterol
MCP-1	Monocyte chemoattractant protein-1
MLR	Monocyte-to-lymphocyte ratio
NCS	Nerve conduction studies
NLR	Neutrophil-to-lymphocyte ratio
OR	Odds ratio
PLR	Platelet-to-lymphocyte ratio
ROC	Receiver operating characteristic
SD	Standard deviation
SNAP	Sensory nerve action potential
SIII	Systemic Immune-Inflammation Index
SIRI	Systemic inflammation response index
T1DM	Type 1 diabetes mellitus
T2DM	Type 2 diabetes mellitus
TyG	Triglyceride–Glucose Index
