# Supplementary material for: Metabolic Dysregulation, Inflammation, and Median Nerve Dysfunction in Patients with Type 2 Diabetes Mellitus with Carpal Tunnel Syndrome
Source: Int J Mol Sci. 2026 May 30;27(11):4995. doi: 10.3390/ijms27114995 (PMC13256965; doi:10.3390/ijms27114995)
Supplement: Supplementary file 1 [file ijms-27-04995-s001.zip › ijms-4301329-supplementary.pdf]

Supplementary Material

Metabolic Dysregulation, Inflammation, and Median Nerve Dysfunction in Patients with Type 2 Diabetes Mellitus with Carpal Tunnel Syndrome

Table S1. Baseline characteristics of the study population divided by electrophysiological severity of CTS.

Variable	All Patients (*n* = 123)	No CTS (*n* = 11)	Mild CTS (*n* = 58)	Moderate and severe CTS (*n* = 54)	*p*
Age (years)	69.00 (61.00-75.00)	65 (57.5-74.5)	70.5 (64-75)	69 (59-74)	0.369
Age group					
Under 60, *n* (%)	28 (22.8)	4 (36.3)	8 (13.7)	16 (29.6)	
60-65, *n* (%)	17 (13.8)	2 (18.1)	9 (15.5)	6 (11.1)	0.296
66-70, *n* (%)	21 (17.1)	0 (0)	12 (20.6)	9 (16.6)	
Over 70, *n* (%)	57 (46.3)	5 (45.4)	29 (50)	23 (42.5)	
Female sex, *n* (%)	58 (47.2)	6 (54.54)	32 (55.17)	20 (37.03)	0.138
Male sex, *n*(%)	65 (52.8)	5 (45.46)	26 (44.83)	34 (62.97)	
Height (cm)	166.64 ± 9.06	168.91±5.79	167.05±8.96	165.74±9.72	0.516
Weight (kg)	83.00 (72.00-98.00)	86.18±17.4	82.65±15.67	86.7±15.86	0.386
BMI (kg/m2)	29.75 (26.67-33.46)	27 (26.2-31.8)	28.7 (26.1-33.2)	30.8 (28.1-35.3)	0.096
BMI over 25 (kg/m2)	107 (86.9)	11 (100)	47 (81.03)	49 (90.74)	0.127
Abdominal Circumference (cm)	109.51 ± 13.46	106.9±18.86	107.62±12.97	112.29±12.46	0.191
T2DM duration (years)	12.00 (6.00-20.00)	8 (3-14.5)	11.5 (5-17)	12 (6-20)	0.362
Systolic BP (mmHg)	140.00 (130.00-147.00)	130 (125-140)	137.5 (130-145)	140 (130-150)	0.276
Diastolic BP (mmHg)	80.00 (73.00-86.00)	81.36±6.8	79.29±9.08	79.44±9.11	0.775
Any history of smoking, *n* (%)	51 (41.5)	7 (63.63)	25 (43.1)	19 (35.18)	0.205
Actively employed, *n* (%)	25 (20.3)	1 (9.09)	7 (12.06)	17 (31.48)	0.024
Retired, *n*(%)	98 (79.7)	10 (90.91)	51 (87.93)	37 (68.52)	
Marital status					
Married *n* (%)	84 (68.3)	6 (54.54)	39 (67.24)	39 (72.22)	
Single, *n* (%)	7 (5.7)	1 (9.09)	2 (3.44)	4 (7.4)	0.591
Widowed, *n* (%)	32 (26)	4 (36.36)	17 (29.31)	11 (20.37)	
Nationality					
Romanian, *n* (%)	98 (79.7)	10 (90.9)	45 (77.58)	43 (79.62)	
Hungarian, *n* (%)	19 (15.4)	1 (9.09)	10 (17.24)	8 (14.81)	0.874
Other, *n* (%)	6 (4.9)	0 (0)	3 (5.17)	3 (5.55)	
Income					
Under 390 € , *n* (%)	52 (42.3)	5 (45.45)	21 (36.2)	26 (48.14)	
390-590 €, *n* (%)	65 (52.8)	6 (54.54)	33 (56.89)	26 (48.14)	0.634
Over 590 €, *n* (%)	6 (4.9)	0 (0)	4 (6.89)	2 (3.7)	
Therapy for T2DM					

Oral Antidiabetic Treatment, *n* (%)	107 (87)	9 (81.81)	48 (82.75)	50 (92.59)	0.262
Metformin, *n* (%)	99 (80.5)	8 (72.7)	46 (79.3)	45 (83.3)	0.687
Sulfonylurea, *n* (%)	25 (20.3)	1 (9.1)	10 (17.2)	14 (25.9)	0.326
DPP4-inhibitors, *n* (%)	14 (11.4)	0 (0)	6 (10.3)	8 (14.8)	0.349
SGLT2-inhibitors, *n* (%)	28 (22.8)	2 (18.2)	15 (25.9)	11 (20.4)	0.732
AR GLP-1 oral, *n* (%)	1 (0.8)	1 (9.1)	0 (0)	0 (0)	0.006
AR GLP-1 injectable, *n* (%)	4 (3.3)	0 (0)	3 (5.2)	1 (1.9)	0.5
Insulin treatment, *n* (%)	48 (39)	1 (9.09)	23 (39.65)	24 (44.44)	0.09
Both Insulin and Oral treatment, *n* (%)	41 (33.3)	1 (9.09)	18 (31.03)	22 (40.74)	0.112

Legend: Continuous variables are presented as mean ± SD for normally distributed data, and as median (IQR) for non-normally distributed data. Categorical variables are shown as counts (percentages). CTS− = absent/mild; CTS+ = moderate/severe. Continuous variables with normal distribution were analyzed using the t-student test, whereas non-normally distributed ones were examined with the Mann-Whitney test. Categorical variables were assessed using Pearson’s chi-square test or Fisher's exact test. DPP4=dipeptidyl peptidase-4; SGLT2=Sodium-Glucose Cotransporter 2; AR GLP-1=Glucagon-Like Peptide-1 Receptor Agonists.
